# 紫杉醇通过上调TAP-1, TAP-2以及消除调节性T细胞逆转肺癌免疫逃逸

**DOI:** 10.3779/j.issn.1009-3419.2010.10.13

**Published:** 2010-10-20

**Authors:** 华 钟, 宝惠 韩

**Affiliations:** 200030 上海，上海市胸科医院肺内科 Department of Pulmonary Medicine, Shanghai Chest Hospital, Shanghai 200030, China

**Keywords:** 紫杉醇, 免疫逃逸, 抗原肽转运蛋白, 调节性T细胞, Paclitaxel, Immunologic escape, Transporters antigen processing, Regulatory T cells

## Abstract

**背景与目的:**

化疗是否可以逆转肿瘤的免疫逃逸尚未得到肯定的回答。本研究旨在探讨紫杉醇类抗肿瘤药物紫杉醇参与逆转肺部肿瘤细胞免疫逃逸的机制。

**方法:**

体外实验应用流式单抗TAP-1、TAP-2检测分析3LL细胞株（Lewis肺腺癌细胞）和经紫杉醇5×10^-8^ mol/L处理的3LL细胞株抗原肽转运体（transporters associated with antigen processing, TAP）的表达；体内实验中，3LL细胞经尾静脉注射成瘤，8 d后化疗组小鼠腹腔内注射紫杉醇：0.012 5 mg/只；对照组小鼠腹腔内注射生理盐水；设立空白对照组；21 d后取各组小鼠的肺部组织，制备单细胞悬液，流式三染法检测调节性T细胞（Regulatory T cells, Treg）。

**结果:**

经紫杉醇处理的3LL细胞高表达TAP-1（5.68%±0.65%）和TAP-2（89.54%±4.8%），明显高于3LL细胞组TAP-1（1.93%±0.25%）和TAP-2（67.78%±5.08%）的表达（*P*分别为0.006和0.036）；体内实验中：荷瘤小鼠肺部肿瘤中Treg细胞的表达（25.46%±2.23%）明显高于正常对照组的小鼠（12.46%±1.21%），差异具有统计学意义（*P* < 0.001）；紫杉醇化疗组明显抑制小鼠肺部肿瘤组织中Treg细胞的表达（17.53%±1.24%），与荷瘤小鼠肺部组织的Treg细胞相比，差异具有统计学意义（*P*=0.004）。

**结论:**

化疗药物紫杉醇通过上调肿瘤细胞表面抗原转运肽的表达以及消除肿瘤局部调节性T细胞的产生，可能部分参与了逆转肺癌免疫逃逸的功能。

## Abstract (en)

**Background and objective:**

Until now, there are still some questions that chemotherapy could block cancer immunologic escape or not. The aim of this study is to explore the mechanism of paclitaxel in the blocking of immunologic escape in 3LL bearing mice.

**Methods:**

MHC class I components were detected by FACS antibodies TAP-1 and TAP-2 in 3LL group and 3LL pretreated with 5×10^-8^ mmol/L paclitaxel. C57BL/6 mice were divided into 3 groups: tumor –bearing group, paclitaxel-treating group and tumor-free group. 3LL cells (0.3×10^6^) were injected via tail vein of mice. On day 8, paclitaxel (0.012 5 mg) was injected subcutaneously in the treatment group. On day 21, murine lung cells were suspended. With FACS triple staining, we analyzed expression of Treg in each group.

**Results:**

The expression of TAP-1 (5.68%±0.65%), TAP-2 (89.54% ±4.8%) was remarkably higher in paclitaxel-pretreating 3LL group than that in 3LL bearing mice group with TAP-1 (1.93%± 0.25%), TAP-2 (67.78%±5.08%), *P*=0.006, *P*=0.036 respectively. The expression of regulatory T cells (Treg) in tumor bearing mice was significantly higher (25.46%±2.23%) than that of in normal mice (12.46%±1.21%) (*P* < 0.001). Expression of Treg in paclitaxel treated mice was remarkably inhibited (17.53%±1.24%) compared with that in 3LL bearing mice group (*P*=0.004).

**Conclusion:**

Paclitaxel may block immunologic escape in the treatment of 3LL bearing mice partly through up-regulation TAP-1, TAP-2 and eliminatiing Treg cells.

化学疗法作为肺癌常规治疗的中坚力量，其目的是最大限度的杀伤肿瘤，但由于化疗药物对机体免疫系统有一定程度的杀伤或抑制效应，因而普遍认为化疗抑制了机体的免疫功能。但是新近的研究发现，某些化疗药物可以通过增强肿瘤细胞免疫原性，促进肿瘤细胞释放诸如热休克蛋白，钙结合蛋白等内源性危险信号，以及抑制调节性T细胞（regulatory T cells, Treg）等机制介导抗肿瘤免疫，对机体的免疫系统有独特的贡献^[1, 2]^；本文研究抗微管的化疗药物—紫杉醇对小鼠肺部肿瘤治疗过程中独特的增强免疫原性的机制；在已有的研究^[3]^中，发现紫杉醇5×10^-8^mol/L的浓度刺激抗原递呈细胞的功能成熟；在本研究中，进一步应用此浓度的紫杉醇探讨其对肿瘤细胞抗原肽转运体（transporters associated with antigen processing, TAP）的表达影响和对调节性T细胞的作用。

## 1 材料与方法

### 1.1 试剂

RPMI-1640，小牛血清为美国Gibco公司产品；重组鼠源性GM-CSF、IL -4为Peprotech公司产品；抗鼠FITC-CD11^b^、PE-CD11^b^、APC-B220、FITC-CD4、PEFoxP3由Biolegend公司提供；流式一抗：FITC-MB1、FITC-Deta、FITC-Calreticulin、FITC-TAP1、FITC-TAP2、FITC-β2microglobulin由Biotechnologies公司提供；流式二抗由DAKO公司提供；小鼠Lewis肺癌细胞株（3LL）为中科院细胞研究所提供；雄性C57BL/6小鼠由上海市肿瘤研究所提供；紫杉醇由美国施贵宝公司提供。

### 1.2 检测肿瘤细胞表面TAP-1和TAP-2表达

细胞培养分两组，一组为3LL细胞培养于RPMI-1640完全培养中，置于37 ℃、5%CO_2_培养箱中；5 d后消化贴壁的肿瘤细胞；另一组细胞在5 d贴壁生长后，加入终浓度为5×10^-8^ mol/L的紫杉醇；48 h后收集细胞；两组细胞分别分成细胞悬液7管，每管含单细胞悬液2×10^6^个-5×10^6^个，用含1%牛血清白蛋白（BSA）的PBS洗涤两次，含2%多聚甲醛的PBS室温下固定20 min，洗涤后，悬浮于含0.5%的PBS液，转移至玻璃小管中，微波处理（200瓦）直至液体沸腾，立即转入冰上冷冻，含1%BSA的PBS洗涤一次；将含1%BSA和0.1%皂角苷的PBS渗透样品30 min，分别加入同型对照，流式一抗：FITC-TAP1、FITC-TAP2；室温下30 min；含1%BSA和0.1%皂角苷的PBS洗涤两次，加入羊抗鼠的流式二抗，室温下30 min；含1%BSA和0.1%皂角苷的PBS洗涤两次后，含1%BSA的PBS再洗涤一次，0.5%多聚甲醛固定，检测；本实验重复3次。

### 1.3 小鼠尾静脉接种肿瘤细胞

3LL肿瘤细胞0.3×10^6^个，悬浮于500 μL PBS液中，从小鼠尾静脉中注入；接种后随机将小鼠分入肿瘤组和化疗组；设立空白对照组。

### 1.4 化疗药物的注射

3LL肿瘤细胞小鼠尾静脉接种后8 d，化疗组小鼠腹腔内接种紫杉醇，0.012 5 mg/只，悬浮于500 μL PBS溶液中；肿瘤组小鼠腹腔内注射500 μL PBS溶液；21 d后取化疗组，肿瘤组，空白对照组小鼠的肺部组织；本组实验重复3次。

### 1.5 小鼠肺部组织单细胞悬液的获得

将DNA酶、胶原酶和透明质酸酶混合，取4.5 mL上述的混合酶。取出肺部组织，眼科手术剪将小鼠肺部剪碎，置于混合酶中，37 ℃、5%CO_2_培养箱中孵育20 min取出。研磨，应用孔径为75 μmol/L的细胞过滤器过滤获得小鼠的肺部组织单细胞悬液；将细胞悬液的浓度调整为1×10^6^/mL。

### 1.6 Treg细胞的流式分析

上述的细胞悬液用FACS缓冲液洗涤，洗涤后加入FoxP3固定液，室温下避光孵育20 min，染色缓冲液洗涤后再用FoxP3缓冲液洗涤一次，悬浮细胞于FoxP3缓冲液中，室温避光15 min，洗涤后，分成5管，每管细胞为1×10^6^个，分别加入同型对照，FITC-CD11^b^、PE-CD11^b^、APC-B220、APC-B220+FITCCD4+PE-FoxP3各20 μL，室温避光30 min，染色缓冲液洗涤2次，上机分析；数据检测应用winMDI 2.8软件分析；二维点阵图勾勒CD25、CD4双阳性细胞，在其中确认FoxP3的细胞，即为Treg细胞的比例；

### 1.7 统计学分析

采用SPSS 10.0软件分析，配对*t*检验和方差分析；所测数据用Mean±SD表示，以*P* < 0.05为差异有统计学意义。

## 2 结果

### 2.1 肿瘤细胞表面TAP表达的检测结果

经紫杉醇5×10^-8^ mol/L处理的3LL细胞表面TAP1的表达（5.68%±0.65%）显著高于3LL细胞组（1.93%±0.25%），差异具有统计学意义（*P*=0.006）。经紫杉醇5×10^-8^ mol/L处理的3LL细胞表面TAP2的表达（89.54%±4.8%）显著高于3LL细胞组（67.78%±5.08%），差异具有统计学意义（*P*=0.036）（表 1）。

**1:** 各组小鼠肺腺癌细胞中TAP-1和TAP-2的表达 The expression of TAP-1 and TAP-2 in each group

Group	The expression of TAP1	*P*	The expression of TAP2	*P*
3LL	1.93%±0.25%	0.006	67.78%±5.08%	0.036
3LL-pretreated with paclitaxel	5.68%±0.65%		89.54%±4.8%	

### 2.2 流式分析定义Treg细胞

典型的流式分析Treg细胞见图 1；根据前向散射仪（FSC）和侧向散射仪（SSC）的参数勾勒所需分析的细胞，定义为R1（图 1A）；在R1细胞群中勾勒CD25和CD4双阳性的细胞，定义为R2（图 1B）；在R2细胞群中分析阳性表达FoxP3的细胞，即为Treg细胞（图 1C）。

**1:**
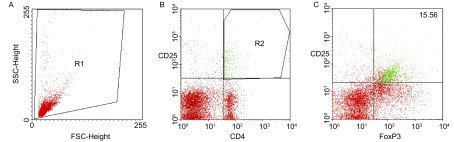
流式分析Treg细胞。根据前向散射仪（FSC）和侧向散射仪（SSC）的参数勾勒所需分析的细胞，为R1（A）；在R1细胞群中勾勒CD25、CD4双阳性的细胞，为R2（B）；在R2细胞群中分析阳性表达FoxP3的细胞，即为Treg细胞（C）。 Analysis Treg cells using FACS. According to the FSC and SSC, R1 (A) was gated; In R1 region, double staining (CD25 and CD4) cells were defined as R2 (B); In R2 region, Treg cells were defined as positive expression of FoxP3 (C).

### 2.3 肺部肿瘤局部Treg细胞的表达

图 2为各组代表性的Treg细胞，图中右上象限的绿色细胞即为CD25+CD4+FoxP3+的Treg细胞。图 2A为正常对照组小鼠肺部的Treg细胞；图 2B为生理盐水治疗的小鼠，其肺部肿瘤细胞中Treg细胞的表达；图 2C为经紫杉醇治疗后的小鼠，其肺癌局部Treg细胞；荷瘤小鼠肺部肿瘤中Treg细胞的表达（25.46%±2.23%）明显高于正常对照组的小鼠（12.46%±1.21%）（*P* < 0.001）；紫杉醇化疗组小鼠肺部肿瘤组织中Treg细胞的表达为17.53%±1.24%，明显低于肿瘤组（25.46%±2.23%）（*P*=0.004）（表 2）。

**2:**
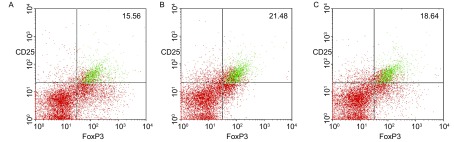
各组一例典型Treg细胞在小鼠肺部组织的表达。A：正常小鼠肺部的Treg细胞（15.56%）；B：生理盐水治疗的肺癌小鼠肺部的Treg细胞（21.48%）；C：紫杉醇治疗的肺癌小鼠肺部Treg细胞（18.64%）。 One typical case of Treg cells expression in each group. A: in lung tissue of normal mice (15.56%); B: 3LL bearing mice pretreated with saline (21.48%); C: 3LL bearing mice pretreated with paclitaxel (18.64%).

**2:** 各组小鼠肿瘤局部的Treg细胞表达的比较 The expression of Treg cells in each group

Groups	The number of Treg cells
Normal mice	12.46%±1.21%
3LL bearing mice pretreated with saline	25.46%±2.23%
3LL bearing mice pretreated with paclitaxel	17.53%±1.24%
The expression of regulatory T cells (Treg) in tumor bearing mice pretreated with saline significantly higher than that of in normal mice (*P* < 0.001). Expression of Treg in mice treated with paclitaxel was inhibited compared with that in 3LL bearing mice treated with saline (*P*=0.004).	

## 3 讨论

TAP是参与肿瘤抗原递呈的重要蛋白，TAP蛋白有两个亚单位组成：TAP1，TAP2。它们负责将细胞内处理完成的抗原肽段转运到内质网。如果肿瘤细胞表面的TAP蛋白表达下降，将会导致肿瘤细胞的主要组织相容性复合物Ⅰ类分子（major histocompatibility complex-I, MHC-Ⅰ）的表达下降或缺如^[4-6]^，结果是肿瘤不呈现或仅呈现弱的抗原性，导致肿瘤抗原无法被递呈给CD8+的T细胞，从而逃脱免疫监管；这是肿瘤免疫逃逸的一个重要机制；而肿瘤免疫逃逸的另一个重要机制是指外周耐受，即调节性T细胞的免疫抑制作用；调节性T细胞（Treg细胞）是一组具有免疫抑制功能的CD4+CD25+T细胞亚群，通过以下机制促进肿瘤的免疫逃避：①通过细胞接触依赖性机制或分泌IL-10、TGF-B的细胞因子抑制免疫细胞的功能；②通过与效应细胞竞争性结合IL-2抑制其增值；③诱导抗原递呈细胞向免疫耐受方向发展^[7]^。

作为肺部肿瘤治疗的重要组成部分——化学治疗，在肺癌的免疫逃逸中发挥什么样的作用成为近年来研究的热点；紫杉醇是目前肺癌化疗中的首选药物；作为抗微管的化疗药物，干扰了微管系统中的动态平衡，使细胞分裂停止在细胞周期的G_2_晚期和有丝分裂期，从而抑制肺癌细胞的复制。本研究试图阐明紫杉醇对于逆转肺癌逃脱免疫监管的内在机制；

我们的研究选择体外5×10^-8^ mol/L紫杉醇的浓度，因为5×10^-8^ mol/L紫杉醇曾经被证实能刺激体内抗原递呈细胞发挥作用^[3]^，对应于体外5×10^-8^ mol/L的紫杉醇，小鼠腹腔内的紫杉醇注射用量是0.012 5 mg/只^[3]^。

本研究指出，紫杉醇具有逆转肿瘤细胞表面降低表达TAP-1、TAP-2蛋白的作用；表现为紫杉醇上调肿瘤细胞表面MHC-Ⅰ类分子的表达。此外，本实验还证实了紫杉醇有利于消除调节性T细胞的抑制作用；Treg细胞的检测在技术上一直是个难点，本研究采用流式三染的技术，将表达CD25、CD4双阳性的细胞再进行细胞内叉头蛋白（FoxP3）的染色。本研究指出，紫杉醇的化疗消除了调节性T细胞在肿瘤免疫中的抑制作用；本研究证明，化疗并不是以往认为的与机体的免疫是对抗或拮抗的；当选择适当剂量的时候，化疗药物可通过促进肿瘤抗原的暴露，消除调节性T细胞促进Th1免疫反应，从而促进抗肿瘤免疫应答的发生；从这点而言，化疗药物对于重建机体的免疫格局具有其独特的贡献。

进一步地，本研究将继续研究化疗药物对于免疫记忆细胞的影响；因为理论上，化疗药物成功杀伤肿瘤细胞，协同免疫系统清除肿瘤抗原，为记忆细胞的分化提供静止期；另一方面，化疗后机体经历淋巴细胞的自稳性增生，使得肿瘤抗原特异性效应细胞的比例大大增加，使更多的细胞分化为相应的记忆细胞^[8, 9]^。

总之，依据化疗后肿瘤细胞免疫原性的变化，设计相应的治疗方案，将化疗和免疫治疗有机的结合，将最终提高抗肿瘤效应，为临床实践带来新思维。
